# Supplementary material for: Tryptophan metabolism determines outcome in tuberculous meningitis: a targeted metabolomic analysis
Source: medRxiv. 2023 Jan 9:2023.01.08.23284316. Preprint. [Version 1] doi: 10.1101/2023.01.08.23284316 (PMC9882445; doi:10.1101/2023.01.08.23284316)
Supplement: Supplement 1 [file NIHPP2023.01.08.23284316v1-supplement-1.pdf]

medRxiv preprint doi: https://doi.org/10.1101/2023.01.08.23284316; this version posted January 9, 2023. The copyright holder for this preprint (which was not certified by peer review) is the author/funder, who has granted medRxiv a license to display the preprint in perpetuity.
It is made available under a CC-BY 4.0 International license .

Supplementary

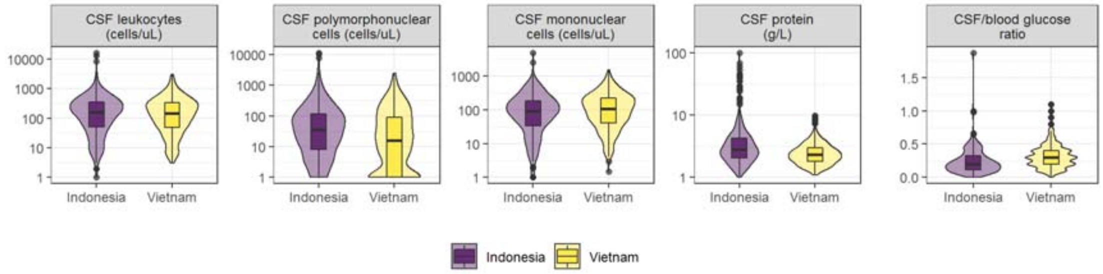

Supplementary Figure 1 CSF parameters of TBM patients in Indonesia and Vietnam. *Distributions of leukocytes, polymorphonuclear cells, mononuclear cells, protein, and the ratio of CSF/blood glucose in Indonesian (purple) and Vietnamese (yellow) patients are depicted by violin plots.*

medRxiv preprint doi: https://doi.org/10.1101/2023.01.08.23284316; this version posted January 9, 2023. The copyright holder for this preprint (which was not certified by peer review) is the author/funder, who has granted medRxiv a license to display the preprint in perpetuity.
It is made available under a CC-BY 4.0 International license .

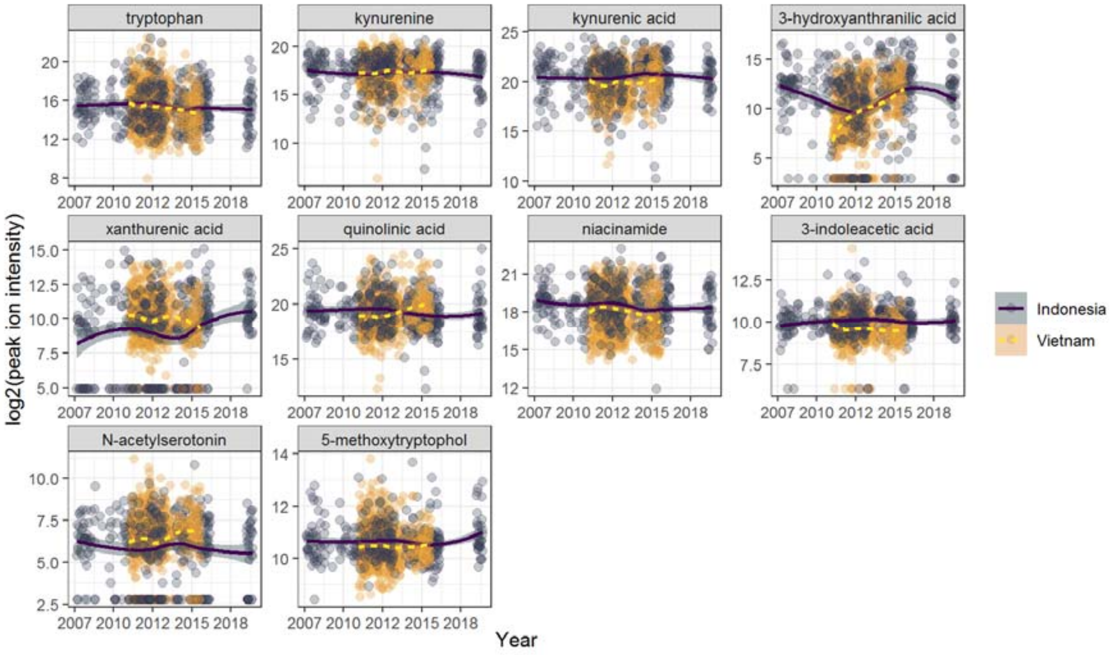

Supplementary Figure 2 Stability of metabolites over-time. *The concentrations of tryptophan metabolites (in log2 scale) were measured in CSF samples from Indonesian (purple) and Vietnamese (yellow) TBM patients were recruited between 2007-2018.*

medRxiv preprint doi: https://doi.org/10.1101/2023.01.08.23284316; this version posted January 9, 2023. The copyright holder for this preprint (which was not certified by peer review) is the author/funder, who has granted medRxiv a license to display the preprint in perpetuity.
It is made available under a CC-BY 4.0 International license .

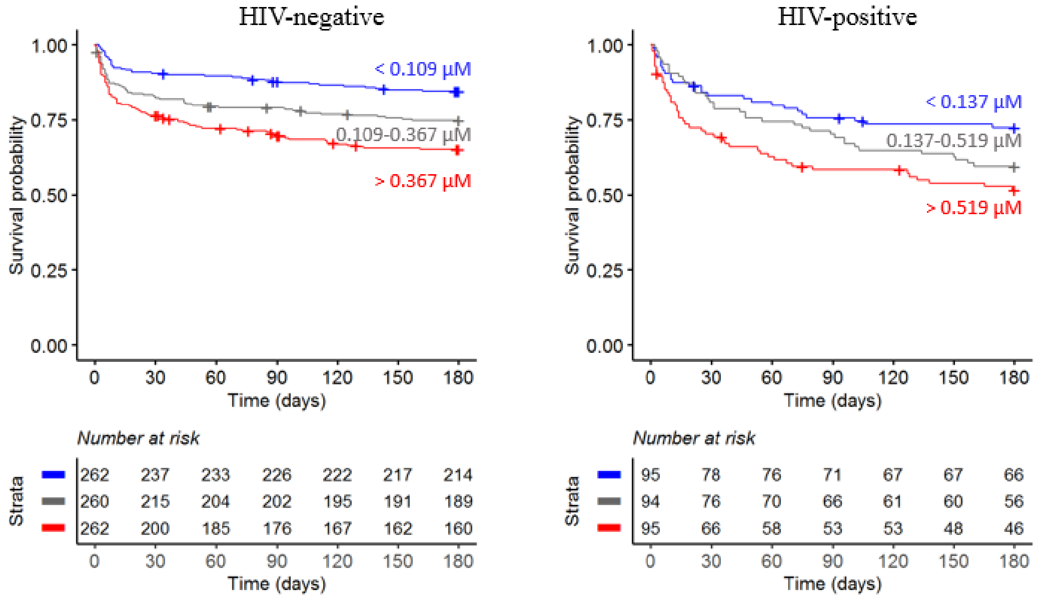

Supplementary Figure 3 Six-month survival curve of TBM patients stratified by HIV status. *Patients were stratified by tertiles based on CSF tryptophan concentrations (red=high tryptophan, gray=intermediate tryptophan, blue=low tryptophan)*

medRxiv preprint doi: https://doi.org/10.1101/2023.01.08.23284316; this version posted January 9, 2023. The copyright holder for this preprint (which was not certified by peer review) is the author/funder, who has granted medRxiv a license to display the preprint in perpetuity.
It is made available under a CC-BY 4.0 International license .

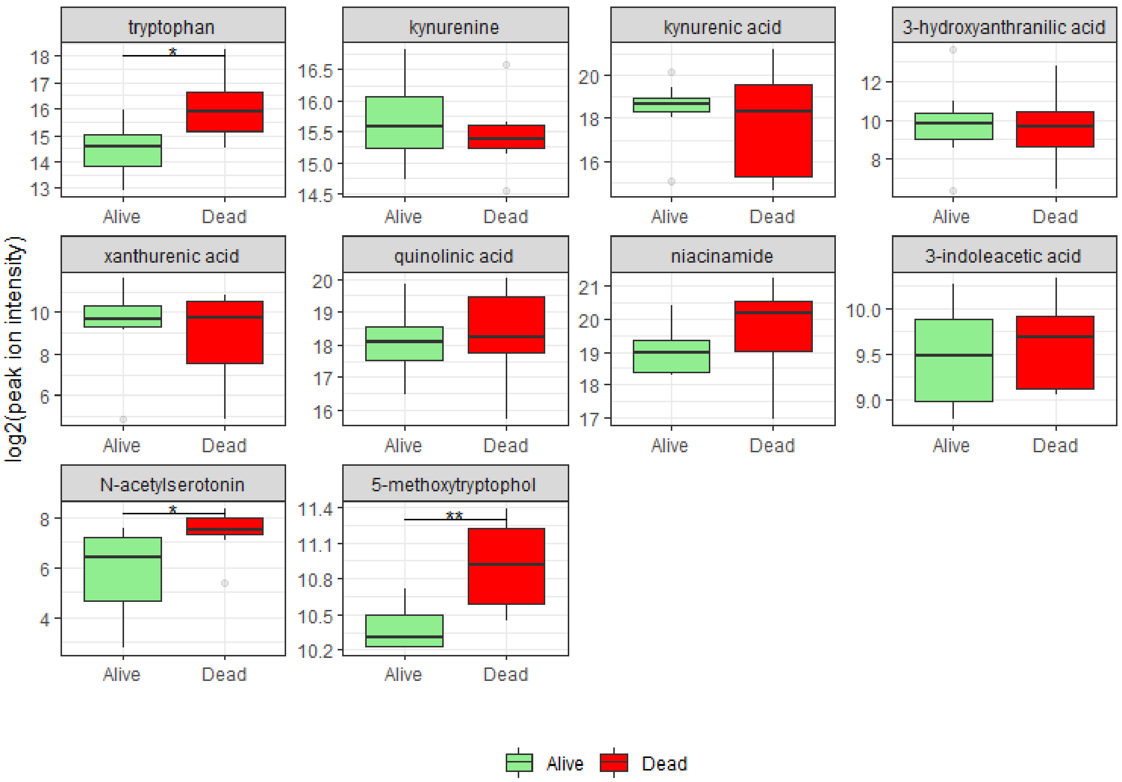

Supplementary Figure 4 In-hospital mortality for 17 HIV-positive patients with cryptococcal meningitis.

medRxiv preprint doi: https://doi.org/10.1101/2023.01.08.23284316; this version posted January 9, 2023. The copyright holder for this preprint (which was not certified by peer review) is the author/funder, who has granted medRxiv a license to display the preprint in perpetuity.
It is made available under a CC-BY 4.0 International license .

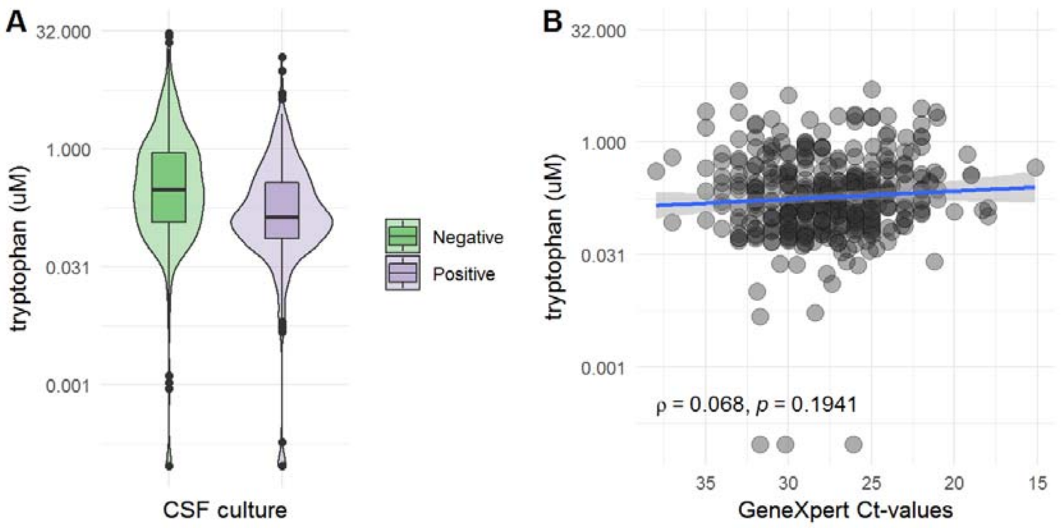

Supplementary Figure 5 CSF tryptophan distributions according to mycobacterial load. *(A) comparing CSF culture negative versus positive patients and (B) among patients with a positive CSF Xpert, in culture positive and culture negative TBM patients, CSF tryptophan was associated with CSF Xpert Ct-values from a low (high Ct-value) to low (low Ct-value) load.*

medRxiv preprint doi: https://doi.org/10.1101/2023.01.08.23284316; this version posted January 9, 2023. The copyright holder for this preprint (which was not certified by peer review) is the author/funder, who has granted medRxiv a license to display the preprint in perpetuity.
It is made available under a CC-BY 4.0 International license .

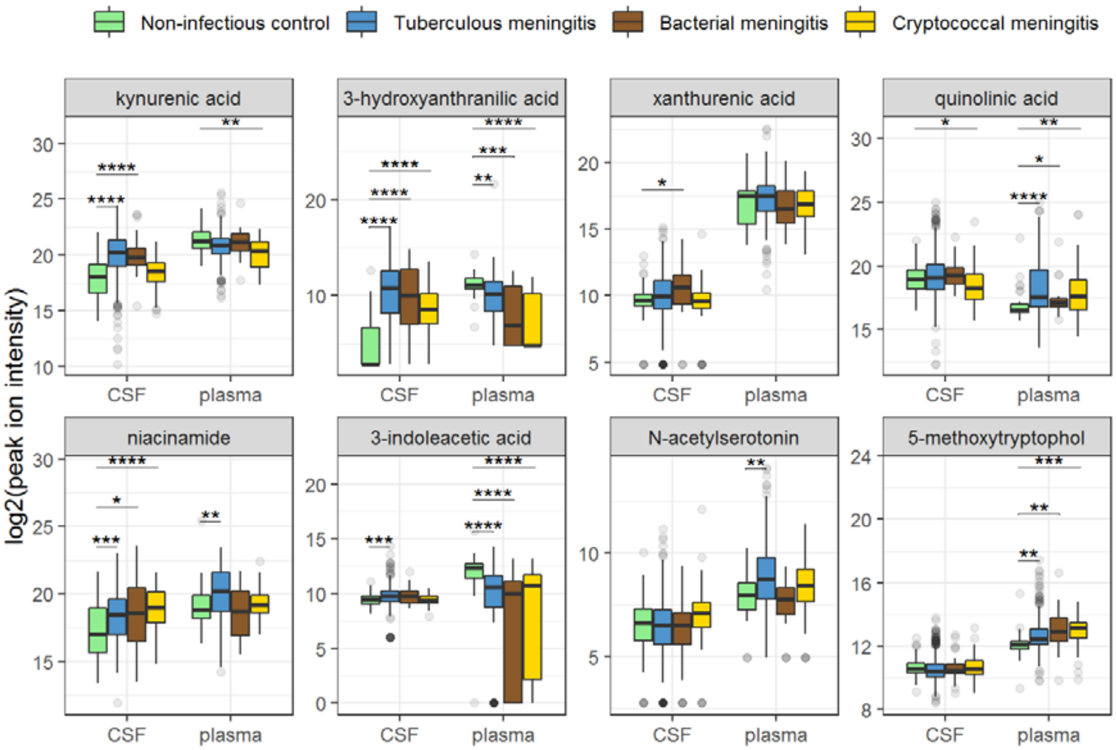

Supplementary Figure 6 Boxplots of CSF and plasma metabolites concentrations in TBM and controls. *Relative concentrations based on peak ion intensities are shown. CSF and plasma concentrations are not directly comparable.*

medRxiv preprint doi: https://doi.org/10.1101/2023.01.08.23284316; this version posted January 9, 2023. The copyright holder for this preprint (which was not certified by peer review) is the author/funder, who has granted medRxiv a license to display the preprint in perpetuity.
It is made available under a CC-BY 4.0 International license .

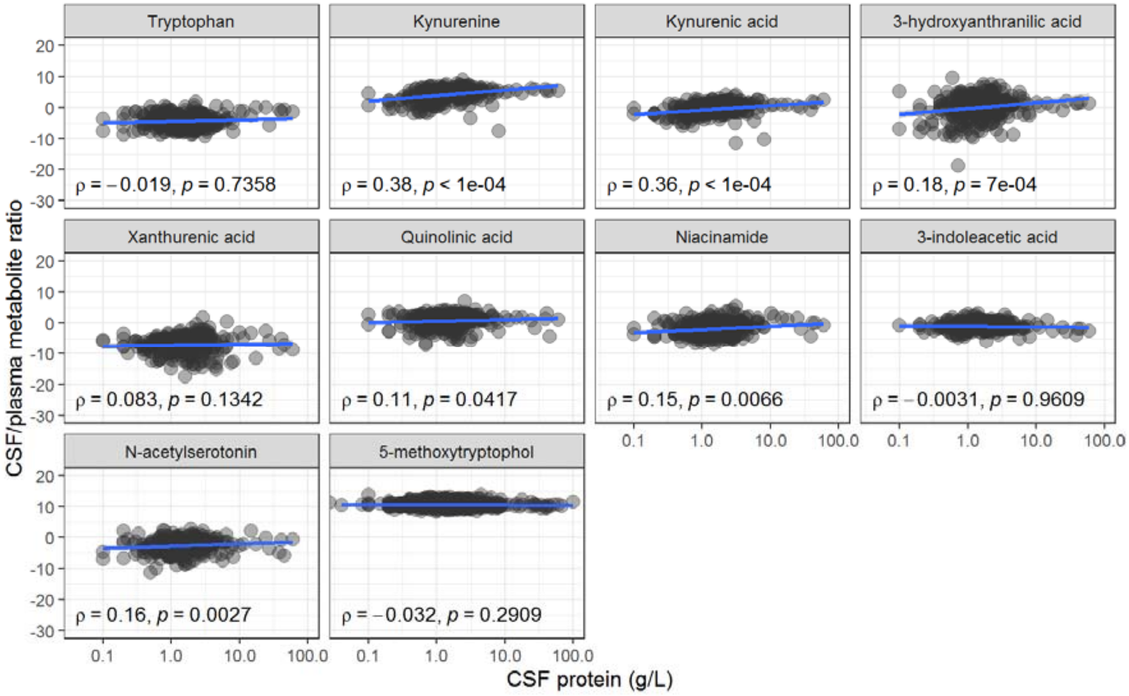

Supplementary Figure 7 Associations between CSF/plasma metabolite ratios (y-axis) and CSF protein levels (as a proxy of CSF barrier leakage, x-axis). *Of note, 71 patients had undetectable plasma levels of 3-indolacetic acid and were removed from this graph.*

medRxiv preprint doi: https://doi.org/10.1101/2023.01.08.23284316; this version posted January 9, 2023. The copyright holder for this preprint (which was not certified by peer review) is the author/funder, who has granted medRxiv a license to display the preprint in perpetuity.
It is made available under a CC-BY 4.0 International license .

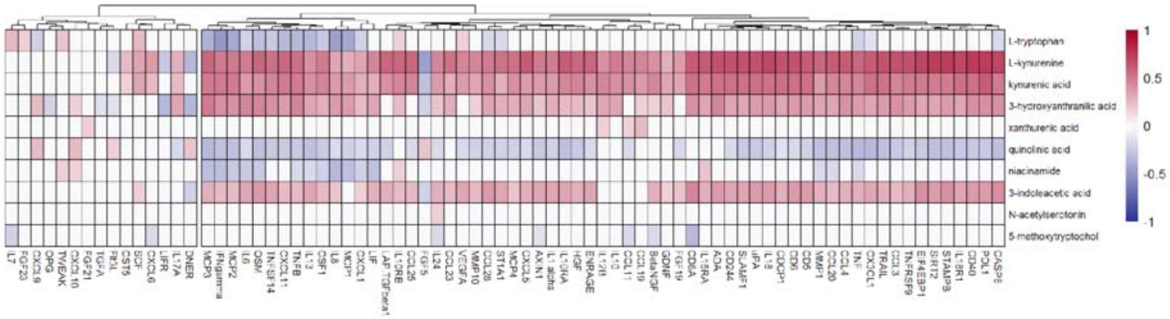

Supplementary Figure 8 Correlation between CSF tryptophan metabolites and inflammatory markers measured with O-link. *Inflammatory markers were clustered based on their correlation coefficients using hierarchical clustering. Red indicates positive correlation, and blue indicated negative correlation.*

medRxiv preprint doi: https://doi.org/10.1101/2023.01.08.23284316; this version posted January 9, 2023. The copyright holder for this preprint
(which was not certified by peer review) is the author/funder, who has granted medRxiv a license to display the preprint in perpetuity.
It is made available under a CC-BY 4.0 International license .

Supplementary Table 1 Univariate Cox regression for influence CSF metabolites on early and late mortality

	Early mortality (day 0-14)				Late mortality (day 14-180)			
**Metabolite**	**HR[1]**	**95% CI[1]**	**p-value**	**FDR[2]**	**HR[1]**	**95% CI[1]**	**p-value**	**FDR[2]**
tryptophan	1.14	1.06, 1.23	<0.001	**0.005**	1.17	1.08, 1.26	<0.001	**<0.001**
kynurenine	1.03	0.95, 1.13	0.4	0.6	1	0.91, 1.10	>0.9	>0.9
kynurenic acid	1.05	0.96, 1.14	0.3	0.5	0.95	0.86, 1.04	0.3	0.5
3-hydroxyanthranilic acid	1.02	0.97, 1.06	0.5	0.6	1.01	0.96, 1.06	0.6	0.7
xanthurenic acid	0.96	0.90, 1.03	0.2	0.5	0.96	0.89, 1.04	0.3	0.5
quinolinic acid	0.89	0.81, 0.98	0.02	0.1	0.9	0.81, 1.00	0.052	0.2
niacinamide	1.02	0.92, 1.12	0.7	0.7	1.05	0.95, 1.16	0.3	0.5
3-indoleacetic acid	1.1	0.92, 1.32	0.3	0.5	1.18	0.97, 1.43	0.093	0.2
N-acetylserotonin	1.05	0.96, 1.14	0.3	0.5	0.96	0.88, 1.06	0.4	0.5
5-methoxytryptophol	1.09	0.87, 1.35	0.5	0.6	1.29	1.04, 1.59	0.02	0.1

Cox regression models were stratified by sites and adjusted by age, sex, and GCS. [1] HR = Hazard Ratio, CI = Confidence Interval. [2] Benjamini & Hochberg correction for multiple testing.

Supplementary Table 2 Univariate Cox regression for influence of plasma metabolites on 60-day mortality

**Metabolite**	**HR[1]**	**95% CI[1]**	**p-value**	**FDR[2]**
Tryptophan	0.8	0.56, 1.16	0.2	0.4
Kynurenine	1.54	1.22, 1.93	<0.001	**0.002**
kynurenic acid	1.2	1.01, 1.43	0.036	0.2
3-hydroxyanthranilic acid	1.13	1.00, 1.28	0.045	0.2
xanthurenic acid	1.11	0.98, 1.25	0.1	0.3
quinolinic acid	0.99	0.88, 1.12	0.9	0.9
Niacinamide	0.92	0.79, 1.07	0.3	0.4
3-indoleacetic acid	1.04	0.97, 1.12	0.3	0.4
N-acetylserotonin	1.05	0.91, 1.22	0.5	0.6
5-methoxytryptophol	1.04	0.85, 1.28	0.7	0.8

Plasma tryptophan metabolites were measured in a subset 300 patients. Cox regression models were stratified by sites and adjusted by age, sex, and GCS. [1] HR = Hazard Ratio, CI = Confidence Interval. [2] Benjamini & Hochberg correction for multiple testing.
